# Encephalopathy associated with propofol infusion syndrome: A case report

**DOI:** 10.1097/MD.0000000000009521

**Published:** 2018-01-05

**Authors:** Xiaolin Li, Zhangning Zhao, Xiaomin Liu, Gaoting Ma, Mei-Jia Zhu

**Affiliations:** a Department of Critical Care Medicine; b Department of Neurology, Affiliated Qianfoshan Hospital of Shandong University, Jinan, Shandong, China.

**Keywords:** encephalopathy, magnetic resonance imaging, propofol, propofol infusion syndrome

## Abstract

**Introduction:**

Propofol infusion syndrome (PRIS) is a rare but potentially fatal complication of propofol infusion. It is clinically characterized by metabolic acidosis, refractory bradycardia, rhabdomyolysis, renal failure, hyperlipidemia, and hepatomegaly. Brain lesion was only reported once in a pediatric patient. We present the 1st adult case with colon polyp and cancer who was diagnosed with PRIS. Her brain magnetic resonance imaging (MRI) and computed tomography (CT) scans reveal prominent bilateral brain lesions, matching with the proposed pathophysiologic mechanism of the syndrome. The patient received prompt acidosis correction and cardiorespiratory support. At last, she died from refractory circulatory failure.

**Conclusion:**

It may be necessary to order a prompt neuroimaging examination in patients suspected with PRIS to judge whether brain lesions exist or not.

## 1 Introduction

Propofol infusion syndrome (PRIS) is a rare but often fatal complication of propofol infusion when the rate exceeds 4 mg/kg hour and/or the duration of use exceeds 48 hours. The syndrome is clinically characterized by metabolic acidosis, refractory bradycardia, rhabdomyolysis, renal failure, hyperlipidemia, and hepatomegaly.^[1–10]^ Occurrence of brain lesions, however, receives little attention. Herein, we present an adult patient with PRIS showing bilateral symmetric brain lesions on computed tomography (CT) and magnetic resonance imaging (MRI), promoting the necessity of neuroimaging in such patients. This study was approved by the local ethics committee, and a written informed consent has been obtained from relatives for publication of this case report and any accompanying image.

## 2 Case presentation

A 30-year-old woman with colon polyp and cancer and a family history of this disease underwent a laparoscopic surgery under sedation with persistent propofol infusion at an average hourly rate of 3.3 mg/kg (120 mg propofol IV for anesthesia induction). The procedure went well until a mild unpredictable hypotension (90/60 mm Hg) was noted 3.5 hours later. Dopamine infusion was initiated for blood pressure support. An immediate capillary glucose measurement was 3.2 mmol/L. Following arterial blood gas measurement revealed a metabolic acidosis (pH 6.91, pCO_2_ 44 mm Hg, pO_2_ 104 mm Hg, base excess −22.4 mmol/L, lactate 15 mmol/L, blood glucose 2.1 mmol/L). Therefore, the patient was treated with sodium carbonate intravenously. Arterial blood gas remeasurement after 15 minutes revealed a pH of 7.28, a lactate of 15 mmol/L, a base excess of −3.1 mmol/L, and a blood glucose of 1.3 mmol/L (normal 3.9–11.1). The patient was immediately given 40 mL of 50% glucose IV followed by constant 5% glucose infusion intravenously. Intermittent arterial blood gas measurement in the next few hours showed pH ranging from 7.06 to 7.49, blood glucose ranging from 11.0 to 12.2 mmol/L, and a persistent high level of lactate equal to or greater than 8.8mmol/L. The propofol infusion was ceased 6 hours after its onset. The patient had remained in an unresponsive state for 3 hours since the propofol discontinuation. Neurological examinations at the moment revealed a gazing toward right and bilateral positive Babinski signs. Therefore, a brain damage was suspected.

**Figure 1:**
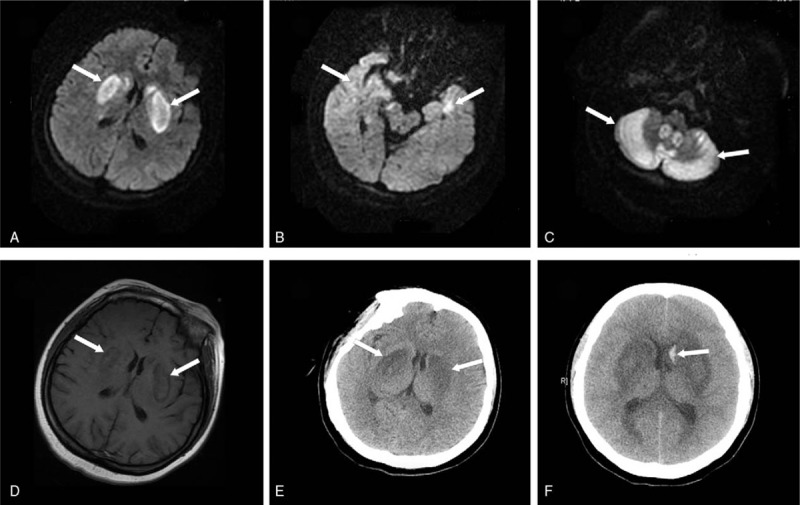
Brain magnetic resonance imaging and computed tomography (CT) scans. Diffusion-weighted imaging shows bilateral hyperintensity lesions of basal ganglia (A, arrows), temporal lobe (B, arrows), and cerebellum (C, arrows), with corresponding hypointensity lesions of basal ganglia on T1-weighted image (D, arrows). CT scan shows decreased attenuation of bilateral basal ganglia (E, arrows). And a repeat CT scan 3 days thereafter shows bleeding of the left caudate nucleus (F, arrow).

Following MRI of the head demonstrated hyperintense signals of the basal ganglia, temporal lobe, and cerebellum bilaterally on diffusion-weighted image (Fig. 1A–C) and a corresponding hypointense signals on T1-weighted image (Fig. 1D). And a CT scan of the head revealed prominent decreased attenuation in the corresponding basal ganglia (Fig. 1E). A repeat CT scan after 3 days revealed bleeding of the left caudate nucleus (Fig. 1F).

To receive better medical therapy for cerebral resuscitation, the patient was transferred to our hospital. Creatine kinase 10 hours after the event onset increased to 1644 IU/L (normal 30–135). Two days later an elevated brain natriuretic peptide (1270 pg/mL, normal <100) was noted, suggesting a heart failure. Cardiac monitoring revealed neither arrhythmia nor hypoxia throughout the procedure. Combination of the lactic acidosis, the elevated creatine kinase level and the cardiac insufficiency in the context of propofol anesthesia suggested PRIS. Considering the probable association between PRIS and mtDNA mutations proposed before,^[11]^ a whole mitochondrial DNA sequencing with Sanger method was conducted and revealed no abnormalities. The patient's relatives refused further investigations. Unfortunately, the patient died due to refractory circulatory failure 16 days following admission.

## 3 Discussion

Propofol is widely used in anesthesia and intensive care due to its short-acting pharmacokinetics, making continuous intravenous infusion easily and facilitating the weaning of patients from ventilator support. However, many serious adverse effects (so called PRIS^[3]^) have been reported to be associated with propofol infusion since the 1st case of death,^[5]^ including refractory bradyarrhythmia, metabolic acidosis, cardiac failure, rhabdomyolysis, renal failure, and hepatomegaly.^[1–10]^ In our patient, metabolic acidosis, cardiac failure, and mild rhabdomyolysis were present during or after the propofol infusion, which cannot be attributed to other causes. The diagnosis of PRIS was made.

Although the mechanisms behind this syndrome have not been fully understood, it is generally accepted that propofol-induced inhibition of intracellular energy production could account for PRIS.^[10]^ In 2001, elevated total carnitine, C5-acylcarnitine, and malonylcarnitine were found in a pediatric patient with PRIS.^[12]^ Malonylcarnitine inhibits the membrane transport protein carnitine palmityl transferase I and entry of long-chain acylcarnitine into mitochondria decreases.^[10]^ Some evidence from animal models show that propofol directly disrupts electron flow down the mitochondrial respiratory electron transport chain.^[13,14]^ In these ways, propofol impairs adenosine triphosphate production in the cell. Thus, tissue with high metabolic activities, such as myocardium and brain are, in theory, more prone to be affected in the clinical setting. Arrhythmia or cardiac dysfunctions were, as mentioned above, frequently observed in patients with PRIS previously,^[1]^ whereas neuroimaging findings were only reported once in a 3-year-old girl with transient limb weakness by Poretti et al,^[15]^ possibly because neuroimaging had not been conducted in patients with nonneurological deficits. MRI of brain in our patient demonstrates prominent bilateral lesions in basal ganglia, temporal lobe, and cerebellum. Etiologies for bilateral basal ganglia MRI abnormalities include hypoxic-ischemic, toxic and metabolic, infectious, immune-mediated, mitochondrial, and neurodegenerative disorders.^[16,17]^ Encephalopathy of hypoxic-ischemic origin could be certainly ruled out in this patient because of the nearly normal degree of blood oxygen saturation and blood pressure throughout the procedure. Considering the acute-onset form, an infectious (eg, Creutzfeldt–Jakob disease, flavivirus encephalitides, and toxoplasmosis) or neurodegenerative (eg, Huntington disease, neurodegeneration with brain iron accumulation, and Fahr disease) lesion would be unlikely. A minority of patients with Behçet disease could manifest initially in the central nerves system, commonly involving basal ganglia,^[18,19]^ but the moderate to strong hyperintense signals on diffusion weighted image argue against the diagnosis.^[20]^ Based on the consideration mentioned above, we suspected a metabolic and toxic etiology. Some toxic poisoning (carbon monoxide, methanol, and cyanide), osmotic myelinolysis, Wernicke encephalopathy, and Leigh disease should be taken into account, but the absence of relevant clinical information make these diagnoses unlikely. Hypoglycemia was a candidate causative factor for the encephalopathy. However, cerebellum is usually unaffected under hypoglycemic status due to the greater activity of the glucose transport mechanisms therein.^[21]^ Moreover, it is unexpected that so short a duration (about 15 minutes) of hypoglycemia could cause so serious brain lesions. For such reasons, we determined the association between the encephalopathy and PRIS.

Some risk factors for PRIS have been proposed, including young age, propofol dosage over 4 to 5 mg/kg per hour, use of duration over 48 hours, critical illness, high fat low carbohydrate intake, inborn error of fatty acid oxidation, and concomitant catecholamine or steroid use.^[10]^ This 30-year-old patient was otherwise healthy except for colon polyp and cancer. No catecholamine or steroid was administered prior to the event. Considering the strong association between PRIS and propofol infusion at doses greater than 4 mg/kg/hour of longer than 48 hours duration,^[3]^ it is confusing that this patient receiving propofol infusion at an average hourly rate of 3.3 mg/kg for about 6 hours could develop the syndrome. We presumed a potential mitochondrial disease in the patient. However, a whole mitochondrial DNA sequencing with Sanger method revealed no abnormalities. Some other predisposing factors might exist and need further investigations.

There is no established standard treatment for PRIS. A sufficient carbohydrate intake might work to prevent PRIS by suppressing the switch to fat metabolism.^[12]^ Wolf et al^[12]^ suggested a carbohydrate intake of 6 to 8 mg/(kg min) in critically ill children. Early recognition plays an important role in successful management of PRIS. The propofol infusion must be stopped immediately. Then hemodialysis or hemoperfusion combined with cardiorespiratory support is advocated.^[22,23]^ Hemodialysis or hemofiltration could decrease metabolic acids and lipids in the blood,^[1]^ and thus to facilitate maintaining hemodynamic stability and alleviate further fat metabolism. Culp et al^[22]^ reported a successful treatment of PRIS with early circulatory support via use of extracorporeal membrane oxygenation. Despite the correction of acidosis and cardiorespiratory support, our patient had remained in a coma, probably due to the profound brain damage. And she died of refractory circulatory failure ultimately. In contrast, Poretti's patient who had basal ganglia spared on MRI recovered completely.^[15]^ It is possible that, like observations in hypoglycemic encephalopathy,^[24]^ basal ganglia involvement in PRIS also indicates a poor outcome.

## 4 Conclusions

We recommend neuroimaging for unresponsive patients with suspected PRIS, because a timely treatment for brain protection may improve the prognosis if brain damage occurs.
